# Supplementary material for: MnO2@Corncob Carbon Composite Electrode and All-Solid-State Supercapacitor with Improved Electrochemical Performance
Source: Materials (Basel). 2019 Jul 26;12(15):2379. doi: 10.3390/ma12152379 (PMC6695780; doi:10.3390/ma12152379)
Supplement: Supplementary file 1 [file materials-12-02379-s001.pdf]

materials

Supplementary Materials: MnO_2@Corncob Carbon Composite Electrode and All-Solid-State Supercapacitor with Improved Electrochemical Performance

Xinsheng Li, Manman Xu, Yang Yang, Quanbo Huang, Xiaoying Wang, Junli Ren and Xiaohui Wang

Calculation of Specific Capacitance and Energy/Power Density

1. *Single Electrode in the Three-Electrode Cell Configuration*

The area-specific capacitances of electrodes were all calculated from their GCD profiles uisng the following equations:

$$C\ (\text{areal}) = \frac{I * \Delta t}{S * \Delta V}$$

where I is the discharge current, Δt is the discharge time, S is the effective area of the electrode, and ΔV is the potential window of the discharge process.

2. *All-Solid-State ASC Device*

The areal specific capacitances of ASC were calculated from its GCD profiles according to following equations:

$$C\ (\text{areal}) = \frac{I * \Delta t}{S * \Delta V}$$

where I is the discharge current, Δt is the discharge time, S is the effective area of the electrode, and ΔV is the voltage difference from the beginning to the end of an individual discharge cycle.

The detailed calculation of energy (E) and power density (P) were as follows:

$$E = 0.5 * C * \Delta V^2$$

$$P = \frac{E}{\Delta t}$$

where C is the area-specific capacitance of the ASC device, Δt and ΔV are the discharge time and potential difference of an individual discharge cycle, respectively.

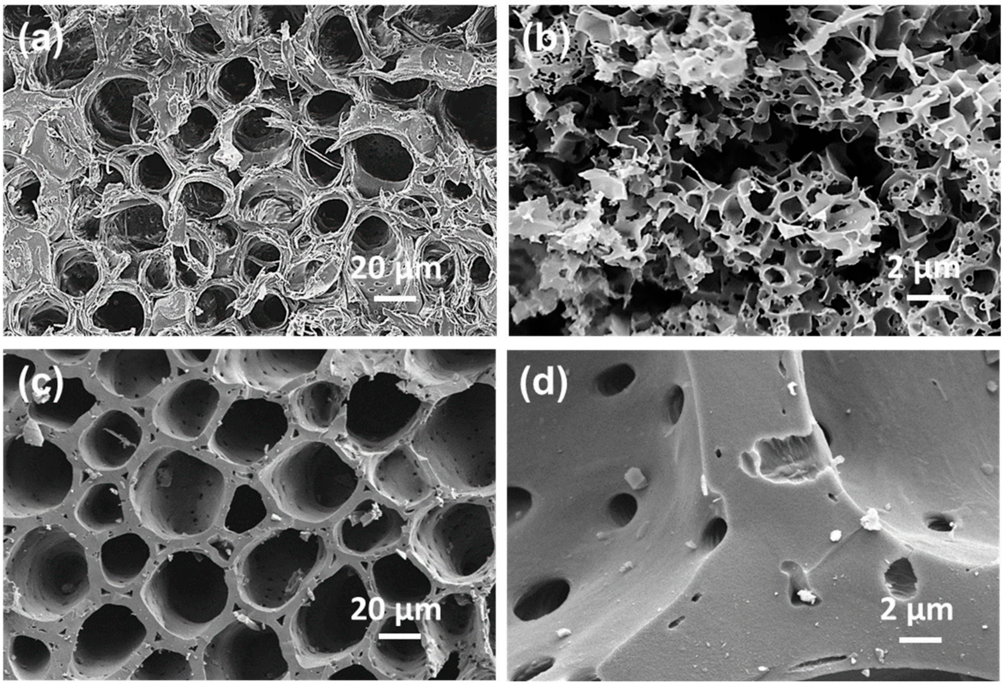

Figure S1. SEM images of the natural corncob (**a**), AC (**b**), and different magnified images of CC (**c**,**d**).

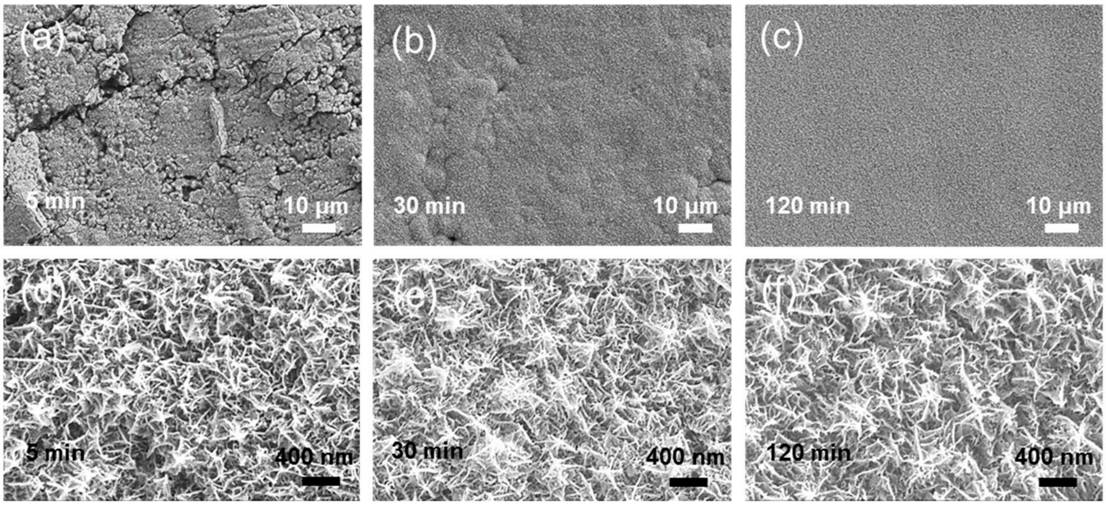

Figure S2. (**a**–**c**) SEM images of MnO2@AC with different electrodeposition times: 5 min (a), 30 min (b), and 120 min (c). (**d**–**f**) HR-SEM images of MnO2@AC with different electrodeposition times: 5 min (d), 30 min (e), and 120 min (f).

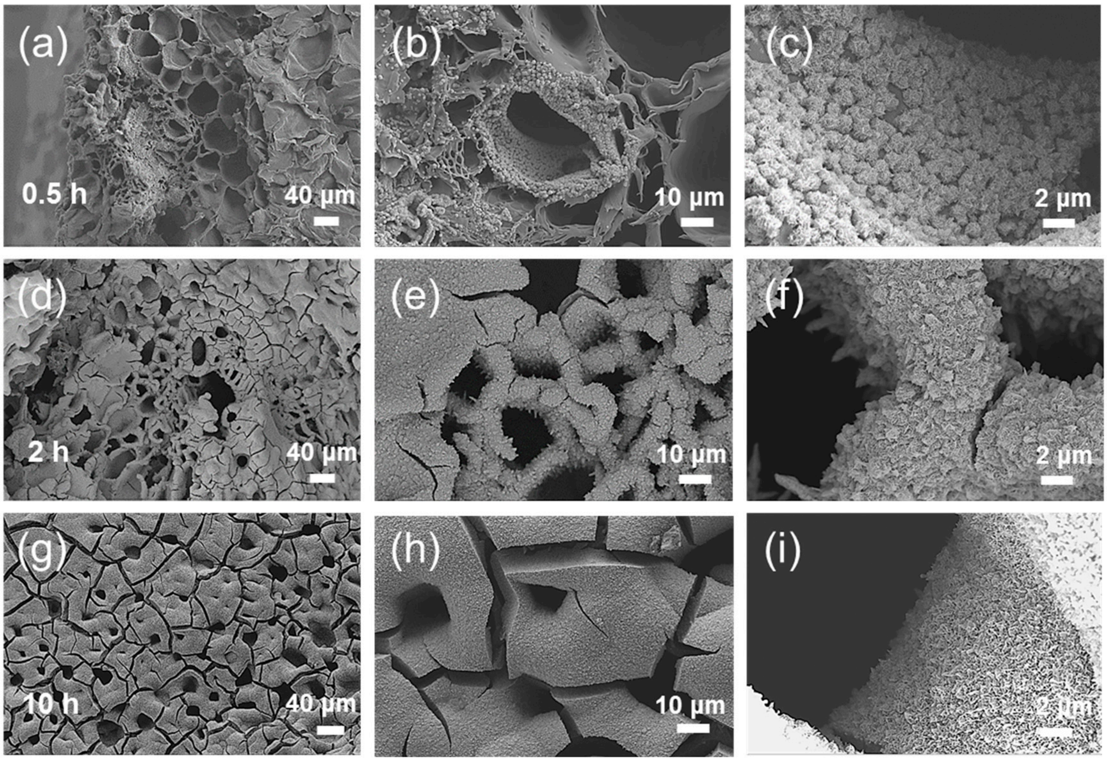

Figure S3. SEM images of MnO_2@CC with different deposition time. (**a**–**c**) SEM images of MnO2@CC-0.5 h with different magnification. (**d**–**f**) SEM images of MnO_2@CC-2 h with different magnification. (**g**–**i**) SEM images of MnO_2@CC-10 h with different magnification.

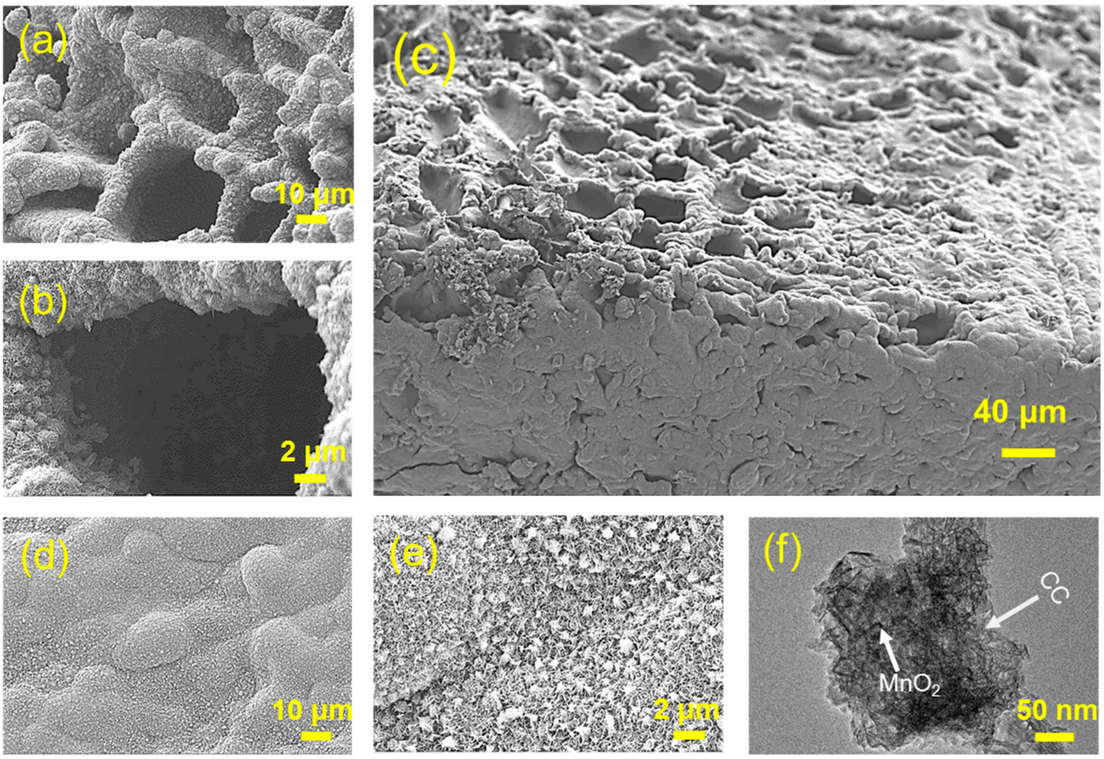

Figure S4. (**a**–**e**) SEM images of MnO_2@CC-5 h. (a) top view, (b) magnified image of (a), (c) cross-sectional view, (d) side view, and (e) magnified image of (d). (**f**) TEM images of MnO_2@CC-5 h.

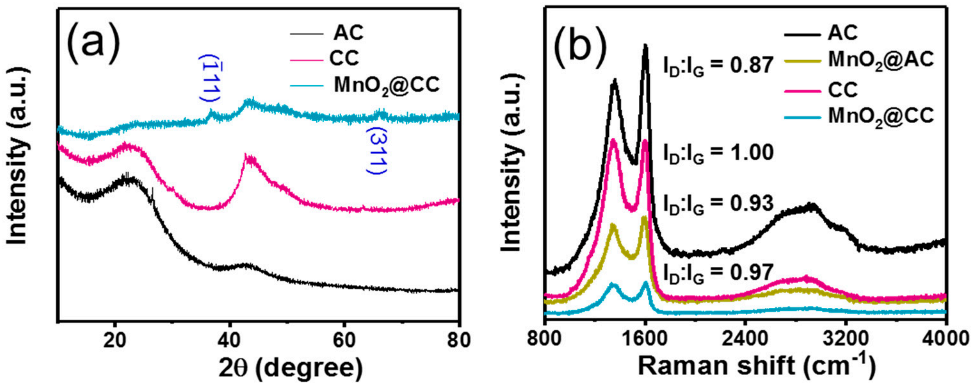

Figure S5. (**a**) XRD patterns of AC and CC-based materials. (**b**) Raman spectrum of corncob-based carbon materials.

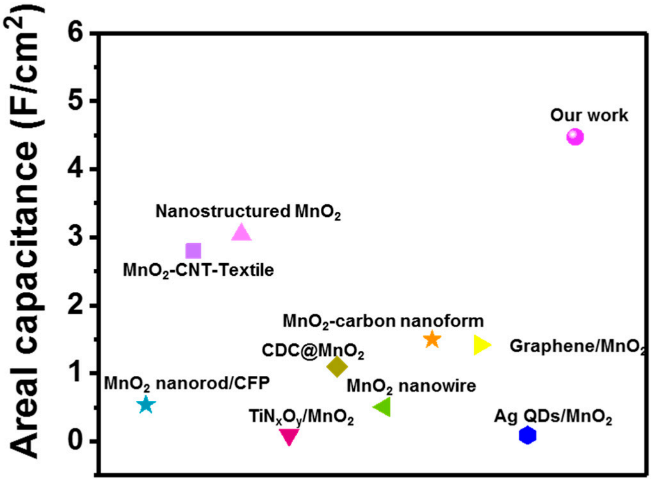

Figure S6. Areal capacitance of MnO_2@CC electrode compared with previously reported electrode materials [1–9].

References:

1. Fischer, A. E.; Pettigrew, K. A.; Rolison, D. R.; Stroud, R. M.; Long, J. W., Incorporation of Homogeneous, Nanoscale MnO_2 within Ultraporous Carbon Structures via Self-Limiting Electroless Deposition: Implications for Electrochemical Capacitors. *Nano Lett.* **2007,** 7, 281–286.
2. He, Y.; Chen, W.; Li, X.; Zhang, Z.; Fu, J.; Zhao, C.; Xie, E., Freestanding Three-Dimensional Graphene/MnO_2 Composite Networks As Ultralight and Flexible Supercapacitor Electrodes. *ACS Nano* **2013,** 7, 174–182.
3. Hu, L.; Chen, W.; Xie, X.; Liu, N.; Yang, Y.; Wu, H.; Yao, Y.; Pasta, M.; Alshareef, H. N.; Cui, Y., Symmetrical MnO_2-Carbon Nanotube-Textile Nanostructures for Wearable Pseudocapacitors with High Mass Loading. *ACS Nano* **2011,** 5, 8904–8913.
4. Huang, Z.-H.; Song, Y.; Feng, D.-Y.; Sun, Z.; Sun, X.; Liu, X.-X., High Mass Loading MnO_2 with Hierarchical Nanostructures for Supercapacitors. *ACS Nano* **2018,** 12, 3557–3567.
5. Lv, Z.; Luo, Y.; Tang, Y.; Wei, J.; Zhu, Z.; Zhou, X.; Li, W.; Zeng, Y.; Zhang, W.; Zhang, Y.; Qi, D.; Pan, S.; Loh, X. J.; Chen, X., Editable Supercapacitors with Customizable Stretchability Based on Mechanically Strengthened Ultralong MnO_2 Nanowire Composite. *Adv. Mater.* **2018,** 30, 1704531.
6. Ye, Z.; Li, T.; Ma, G.; Peng, X.; Zhao, J., Morphology controlled MnO_2 electrodeposited on carbon fiber paper for high-performance supercapacitors. *J. Power Sources* **2017,** 351, 51–57.

7. Zhang, J.; Li, Y.; Zhang, Y.; Qian, X.; Niu, R.; Hu, R.; Zhu, X.; Wang, X.; Zhu, J., The enhanced adhesion between overlong TiN_xO_y/MnO_2 nanoarrays and Ti substrate: Towards flexible supercapacitors with high energy density and long service life. *Nano Energy* **2018,** 43, 91–102.
8. Zhang, X.; Fu, Q.; Huang, H.; Wei, L.; Guo, X., Silver-Quantum-Dot-Modified MoO_3 and MnO_2 Paper-Like Freestanding Films for Flexible Solid-State Asymmetric Supercapacitors. *Small* **2019,** 15, 1805235.
9. Zhang, Y.; Yuan, X.; Lu, W.; Yan, Y.; Zhu, J.; Chou, T.-W., MnO_2 based sandwich structure electrode for supercapacitor with large voltage window and high mass loading. *Chem. Eng. J.* **2019,** 368, 525–532.

© 2019 by the authors. Submitted for possible open access publication under the terms and conditions of the Creative Commons Attribution (CC BY) license (http://creativecommons.org/licenses/by/4.0/).
